# Spatiotemporal Expression of Matrix Metalloproteinases (MMPs) is Regulated by the Ca^2+^-Signal Transducer S100A4 in the Pathogenesis of Thoracic Aortic Aneurysm

**DOI:** 10.1371/journal.pone.0070057

**Published:** 2013-07-29

**Authors:** Jiumei Cao, Liang Geng, Qihong Wu, Wei Wang, Qiujing Chen, Lin Lu, Weifeng Shen, Ying Chen

**Affiliations:** 1 Department of Geratology, Ruijin Hospital, Shanghai Jiaotong University School of Medicine, Shanghai, China; 2 Department of Cardiology, Ruijin Hospital, Shanghai Jiaotong University School of Medicine, Shanghai, China; 3 Department of Cardiology, Ruijin Hospital North, Shanghai Jiaotong University School of Medicine, Shanghai, China; Northwestern University Feinberg School of Medicine, United States of America

## Abstract

**Aims:**

This study investigated whether S100A4 plays a potential role in the formation of thoracic aortic aneurysm (TAA).

**Methods and Results:**

The thoracic aortas of male Sprague-Dawley rats were exposed to 0.5 M CaCl2 or normal saline (NaCl). Animals were euthanized at specified time-points (2, 4, and 10 weeks post-TAA induction). The treated aortic segments were harvested, and mRNA levels, protein expressions and immunohistochemistry of MMP-2, MMP-9 and S100A4 were analyzed. The A7r5 cell lines were used for an in vitro study. Experiments were also performed using human TAA samples for comparison. Localized aneurysmal dilation was observed in the CaCl2-treated segments. The transcription levels of S100A4 and MMPs were elevated in CaCl2-treated segments versus controls, and a significant correlation between S100A4 and expression of MMPs was observed across all time-points. Immunohistochemical studies revealed similar expression pattern of S100A4 and MMP proteins, as well as co-localization of S100A4 with the cell lineage markers (αSMA and CD68) and inflammatory markers (MMPs and NF-κB P65 subunit). The proliferative ability of A7r5 cells after transfection with S100A4 siRNA was suppressed, and down-regulation of S100A4 inhibited MMP-2 and MMP-9 expression in vitro. Increased expression of S100A4 was observed in all layers of the aorta wall in human TAA specimens. Serum concentrations of S100A4 determined by ELISA were found to be significantly increased in TAA patients.

**Conclusions:**

This study established the important roles of S100A4 and MMPs in the development of TAA.

## Introduction

Aortic aneurysms are caused by extensive dilation of a weakened area of the aortic wall due to loss of normal structural integrity [1]. According to the disease site, aortic aneurysms can be generally classified into two major categories: thoracic aortic aneurysm (TAA, occurring in the chest) and abdominal aortic aneurysm (AAA, occurring in the abdomen). Less frequently, aortic aneurysms also occur across both areas and are called thoracoabdominal aortic aneurysms. Since the dilated and over-stretched blood vessel walls are prone to rupture, often leading to sudden internal bleeding and death, aortic aneurysms represent a great risk to human life. Although it is not entirely known why aortic aneurysms occur, accumulated studies have revealed that multiple environmental and genetic risk factors are involved in disease development, such as smoking, high blood pressure, high cholesterol, atherosclerosis, overweight and family history of aneurysms or other known genetic syndromes (e.g. Marfan syndrome, Ehlers-Danlos syndrome) [2].

TAA account for about 20% of aortic aneurysm cases. In contrast to AAA, TAA tend to occur at an earlier age and are relatively more associated with hereditary influences [1]. A common pathogenic feature of TAA is progressive medial degeneration, which is characterized by elastic fiber degeneration, accumulation of proteoglycans, and loss of vascular smooth muscle cells (VSMCs). Less conspicuously, disarrayed nodular proliferation of VSMCs in the subintima [3] or medial area [4] has also been observed in association with specific genetic mutations. The mechanisms underlying such pathological alterations have been extensively studied and many disease-associated genes have been identified: for example, the genes encoding VSMC intracellular contractile proteins [including Myosin heavy chain (MYH11) and α-smooth muscle actin (ACTA2)], the major TGF-beta receptor genes (TGFBR-1 and TGFBR-2), and the genes functioning as extracellular matrix elements such as Fibrillin-1 (FBN1) and Collagen α-1 (COL3A1) [2]. Recent studies have suggested an important role for the matrix metalloproteinase (MMP) proteins, which are proteases capable of degrading extracellular matrix proteins, in vascular remodeling during TAA development. For instance, high levels of MMP expression and activity have been observed in natural and experimentally-induced aneurysms [5]–[7]. Probably the most direct evidence that MMPs can contribute to aneurysm development came from a study on knock-out mice [8] and from studies showing that MMP inhibitors (such as doxycycline) could attenuate aneurysm progression [9], [10]. However, the pathogenic mechanisms leading to the activation of the MMP system in aneurysm development are still poorly defined.

In this article, we report the coincidence of the spatiotemporal expression pattern between MMPs and S100A4, a member of the S100 calcium-binding protein family largely known for its role in cancer cell metastasis [11], identified by our recent CaCl_2_-induced TAA animal study. It was further revealed in our *in vitro* cell culture assays that siRNA-suppression of S100A4 expression could lead to down-regulation of MMP-2 expression. By examining human TAA specimens and control samples, we also observed a significantly increased level of S100A4 expression in the tissues and serum of TAA patients. Taken together, these findings suggest that S100A4 contributes to TAA pathogenesis by functioning, at least partially, as a regulator of MMP expression. Increased expression of S100A4 in the tissue or serum of TAA patients may potentially serve as a biomarker for disease diagnosis and treatment.

## Methods

### Animal Experiment

The experimental study was conducted in accordance with the Guide for the Care and Usage of Laboratory Animals published by the US National Institutes of Health (NIH Publication No. 85–23, revised 1996), and approved by the Animal Protection Committee of Shanghai Jiao Tong University. Male Sprague-Dawley rats (250–300 g) were randomly allocated to the CaCl_2_-treated group and the NaCl-treated group. All rats were examined at 2, 4, and 10 weeks after induction (at each time point, n = 15 in the CaCl_2_-treated group and n = 10 in the NaCl-treated group). Details regarding establishment of the TAA model in rats have been described previously [7]. Briefly, anesthesia was induced by inhalation of isofluorane mix and maintained with continuous flow of 1.5% isofluorane, and then all animals were subjected to orotracheal intubation. A lateral incision was made at the fifth intercostal space to expose the thoracic aorta. The exposed thoracic aortic segment was then wrapped with a strip pre-soaked in 0.5 M CaCl_2_ solution or normal saline for 15 minutes. All rats were allowed to recover and kept in specific pathogen-free conditions under standard temperature and humidity.

### Tissue Isolation

Ketamine-xylazine was used to anesthetize rats prior to euthanasia. Animals were euthanized at 2, 4, and 10 weeks. The thorax was opened beneath the xiphoid process. An incision was made in the right atrium to provide an outlet for blood and perfusate. The cardiovascular system was perfused with normal saline from the left ventricle at a pressure of 100 mmHg for 10 minutes, and the entire aorta was carefully harvested. Aortic tissues were stored at −80°C until RNA isolation. For histological and immunohistochemical studies, the cardiovascular system was subjected to perfusion with 4% formalin for 10 minutes. Aortic segments treated with CaCl_2_ or NaCl were then harvested and stored in 4% formalin solution at 4°C, then embedded in paraffin. Sections of 5 µm thickness were cut and mounted on glass slides.

For the study of human TAA tissue, written informed consent was obtained from all patients and their relatives, and the protocol was approved by the Ruijin Hospital Ethical Committee and followed the principles of the Declaration of Helsinki. Human TAA samples were obtained from patients undergoing TAA surgery. Aortic tissue of the same region was obtained from individuals who died of non-vascular disease and were age- and gender-matched to the patients as closely as possible. The TAA patient tissue was obtained from the surgery. And then aortic segments were stored in 4% formalin solution at 4°C, then embedded in paraffin. The aortic segments of the control group were obtained during performing the autopsy which was examinated within 24 hours after the death. The serum of the TAA patients was obtained before the surgery, and the serum of the control group was obtained from the clinical laboratory.

### Histology

Hematoxylin and eosin (HE) staining was performed for morphometric analysis. Intima-media thickness (IMT) and external media diameter were determined using Image pro plus 6.0 (Media cybernetics). Elastic fiber content was assessed by Weigert staining, and the disruption of elastic fibers was graded by two independent observers on a scale of 1 to 4 (1 = normal or disruption <25%, 2 = disruption 25% to 49%, 3 = disruption of 50% to 75%, 4 = disruption >75% or total absence).

### Immunohistochemistry

Slides were immunostained with various antibodies (**Table S1**) according to standard protocols. The secondary anti-rabbit/mouse HRP/DAB detection system was used to visualize positive expression. For immunofluorescence, the slides were stained with FITC-conjugated anti-mouse secondary antibody (1∶50, Invitrogen) and Alexa Fluor 594-conjugated anti-rabbit secondary Ab (1∶1000 Invitrogen). After washing, the slides were further stained with DAPI. Rabbit or mouse IgG was used as the negative control. For immunohistochemistry, the positively-stained area was determined using threshold-based digital planimetry software (Image pro plus 6.0; Media cybernetics), and expressed as the percentage of the positive area in the intima-media or adventitia.

### Real-time PCR

Total RNA was extracted using RNA iso-plus reagent (Takara) according to the manufacturer’s instructions. RNA was reverse transcribed to cDNA using an RT-PCR kit (Promega). Specific primers were designed based on the respective cDNA sequences, and the β-actin gene was used as an endogenous reference. Real-time PCR reactions were performed using a SYBR Green kit (Takara) in a reaction volume of 50 µL. Each cDNA was amplified in triplicate PCR reactions.

### Cell Culture and Transfection of A7r5 with S100A4 siRNA

A7r5 cells were purchased from the American Type Culture Collection (Manassas, VA). They were routinely maintained in DMEM supplemented with 10% fetal calf serum in a humidified atmosphere of 95% air and 5% CO2 at 37°C. Cells were grown to 80% confluence prior to treatment in the various experiments. The S100A4 siRNA sequences, synthesized by Shanghai GeneChemat Company of China were as follows: sense strand: 5′-gga cag acg aag ctg cat tcc a dTdT-3′, anti-sense strand: dTdT 3′-ccu guc ugc uuc gac gua agg u-5′. Control nonspecific siRNA was purchased from Shanghai GeneChemat Company and the sense strand: 5′-aat tct ccg aac gtg tct cgt-3′, anti-sense strand: 3′-uua aga ggc uug cac aga gca-5′.When cells reached 80–90% confluence, A7r5 cells were transfected with S100A4 siRNA according to the manufacturer’s instructions. In parallel, untreated cells and cells transfected with nonspecific siRNA were used as controls. Experiments were performed in triplicate for each experimental condition.

### Cell Proliferation Assay

DNA incorporation into proliferating cells was quantified using a BrdU Cell Proliferation Kit (Chemicon, Cat. 2752). Briefly, A7r5 cells were seeded in 96-well plates (20 000 cells/well) and grown for 8 h in DMEM containing 10% FBS. The cells were serum-starved for 24 h for cell cycle synchronization. Subsequently, untreated cells, and cells transfected with S100A4 siRNA or nonspecific siRNA were processed according to the protocol. BrdU was then added to the culture medium, and BrdU incorporation was quantified by ELISA according to the manufacturer’s instructions. Experiments were performed in triplicate for each experimental condition.

### Western Blot Analysis

In the cell culture experiment, A7R5 cells were divided into three groups: group one was untransfected, group two was transfected with nonspecific siRNA, group three was transfected with S100A4 specific siRNA. At each time point, cells were washed twice with ice-cold PBS and lysed in RIPA buffer (50 mM Tris, PH 7.5, 150 mM NaCl, 1% NP-40, 0.5% sodium deoxycholate and 0.1% SDS) supplemented with protease inhibitor cocktail (Sigma) and phosphatase inhibitor cocktail(Sigma).Protein concentration was determined by the Bio-Rad protein assay. Total cell lysates(10 µg ) were separated by SDS-PAGE with Tris-HCl gel (Ready Gel, BIO-RAD, Hercules, CA, USA), followed by transfer to polyvinylidene difluoride membranes (Immobilon-P, Millipore, Bedford, MA, USA). Membranes were incubated in blocking buffer (5% nonfat milk in T-PBS) for 1 h and immunoblotted with primary antibody diluted in 5% bovine serum albumin(BSA, Sigma).Membranes were probed with horseradish peroxidase-conjugated secondary antibody (Jackson ImmunoResearch Laboratories, West Grove, PA, USA). Western blots were visualized by the enhanced chemiluminescence technique (Amersham ECL Western Blotting Detection Reagents, GE Healthcare, Piscataway, NJ, USA). Primary antibodies include anti-MMP2, anti-MMP9, anti-GAPDH (all from Cell Signaling Technology).

### Serum S100A4 Concentration

Serum S100A4 concentrations from TAA patients and controls (n = 6 of each) were measured by ELISA. The ELISA system was calibrated with purified recombinant mouse S100A4 (R&D Systems, Inc., Minneapolis, MN) according to the manufacturer’s instructions. The absorbance was then measured at 450 nm.

### Statistical Analysis

The morphometric analysis was performed blinded for the statistician. Every 20th section in each CaCl_2_-treated region was collected to calculate the external media diameter, and the average value from 5 sections was used for each sample. For structural and immunohistological analysis, 3 slides from each sample were analyzed, and 3 high-power images of each slide were randomly selected. The average result of the nine images represented the final value for each sample. Blinded cell counting by two independent observers was completed to determine the percentage of cells with and without positive stain against MMP-2,MMP-9,S100A4 and αSMA. Due to the variability in the number of cells present in the tissue samples, results were calculated as percentage of the cells staining versus the total number of cells. Quantitation of mRNA was performed by the ΔΔCT method, and expressed as fold changes in expression versus reference control samples.

Data are presented as mean ± (SEM). Differences between groups were analyzed using one-way ANOVA, with the post-hoc Dunnet C test. A two-sided probability level of *P*<0.05 was considered statistically significant. All analyses were performed using SPSS for Windows 13.0.

## Results

### Aortic Pathology of CaCl_2_–induced TAA in a Rat Model

**Figure 1:**
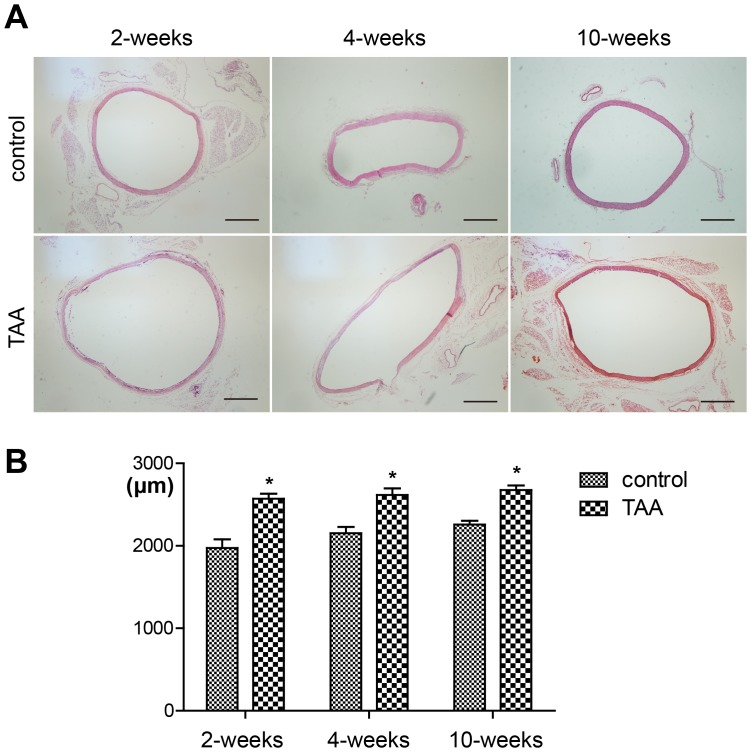
Increased aortic diameter at all time-points after thoracic aortic aneurysm induction. A, Representative lower power micrographs of hematoxylin/eosin-stained, NaCl-treated aorta (control group) and CaCl_2_-treated aorta (TAA group). Scale bar = 500 µm. B, Measurements of external media diameter in the control group and TAA group at all time-points. Bars represent the SEM, *P<0.05 compared with the control group at each time-point (n = 6 for each group).

**Figure 2:**
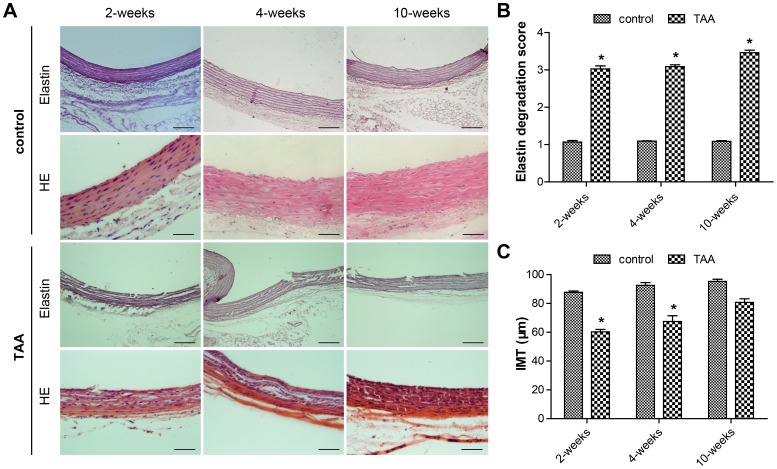
Disruption of the elastic lamellar structure at all time-points after thoracic aortic aneurysm induction. A, Representative Weigert staining and hematoxylin/eosin staining of sections of the aorta from NaCl-treated and TAA-induced rats. Scale bar = 100 µm. B, Grading of elastin degradation at each time-point in the two groups. C, Quantitation of intima-media thickness (IMT) in the two groups. Bars represent the SEM, *P<0.05 compared with the control group (n = 6 for each group).

HE staining showed significant aneurysmal dilation in CaCl_2_-treated segments compared to NaCl-treated segments at all time-points **(** **Fig. 1A** **)**. The external media diameter measurements showed an immediately increase at 2 weeks, which does not further increase at 4 and 10 weeks. **(** **Fig. 1B** **)**. Conspicuous disruption of the elastic lamellar structure in CaCl_2_-treated segments was observed at all time-points **(** **Fig. 2A** **)**, with the most serious medial degeneration occurring at the 10th week after induction **(** **Fig. 2B** **)**. The intima-media thickness (IMT) was significantly decreased at 2 and 4 weeks after CaCl_2_ treatment, compared with that of the controls **(** **Fig. 2C** **)**.

### MMP-2, MMP-9 and S100A4 Expression in Rat Aortic Tissue

**Figure 3:**
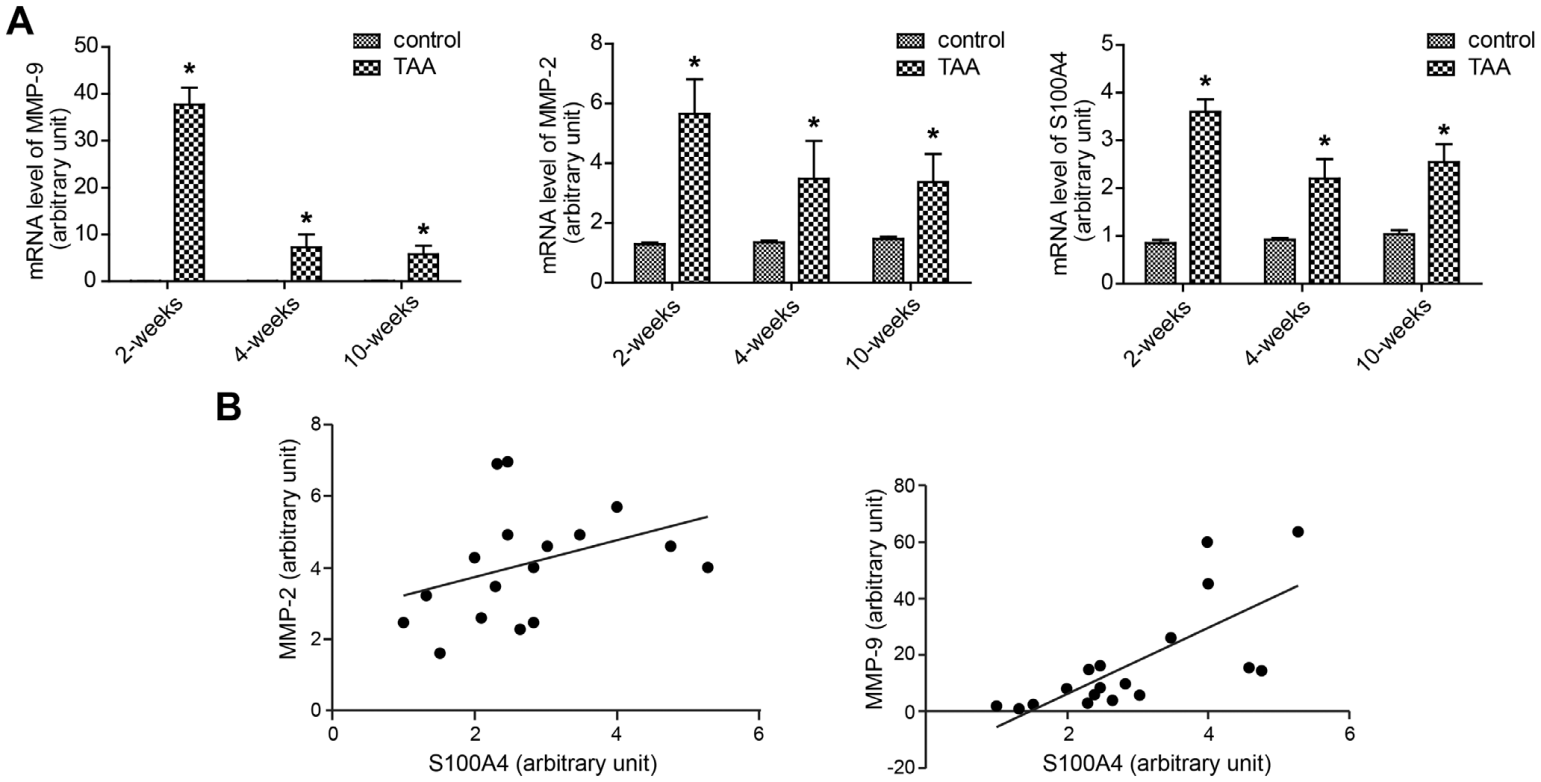
Increased transcription levels of MMP-2, MMP-9 and S100A4A at all time-points after thoracic aortic aneurysm induction. A, Real-time PCR examination of MMP-2, MMP-9 and S100A4 at each time-point in the two groups, and mRNA levels are normalized to the mRNA of *β*-actin. Bars represent the SEM, *P<0.05 compared with control group (n = 6 for each group). B, Positive correlations between the mRNA levels of S100A4 and MMPs over time post-TAA induction.

The transcription levels of MMP-2, MMP-9 and S100A4 were analyzed by real time PCR and are summarized in **Fig. 3A**. Both MMP-2 and MMP-9 mRNA levels were found to reach the highest level at 2 weeks post-TAA induction, followed by a gradual decrease at the 4- and 10-week time-points but still remaining higher than those of the NaCl-treated control group. S100A4 mRNA levels followed a remarkably similar pattern to those of MMP2 and MMP9 **(** **Fig. 3A** **)** and a positive correlation in transcript expression was established between S100A4 and both MMP-2 and MMP-9 (MMP-2, r = 0.652, *P*<0.05; MMP-9, r = 0.762, *P*<0.05) (**Fig. 3B**).

**Figure 4:**
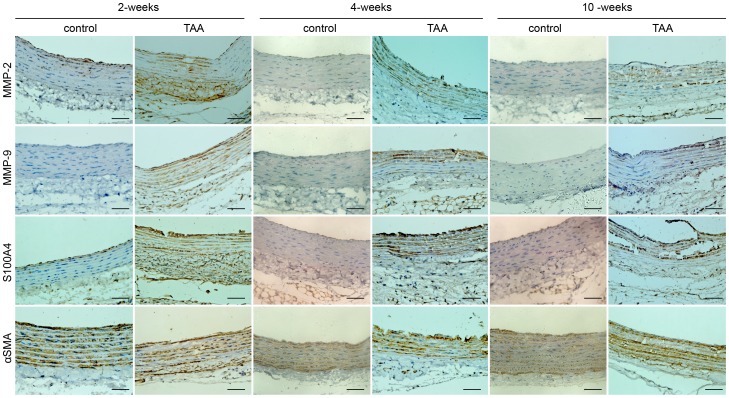
Representative pictures of immunohistochemical staining in aortic slides over time post-TAA induction. The slides were stained with antibodies against MMP-2, MMP-9, S100A4 and αSMA. An anti rabbit HRP/DAB detection system was used to visualize expression (brown staining). Scale bar = 50 µm.

**Figure 5:**
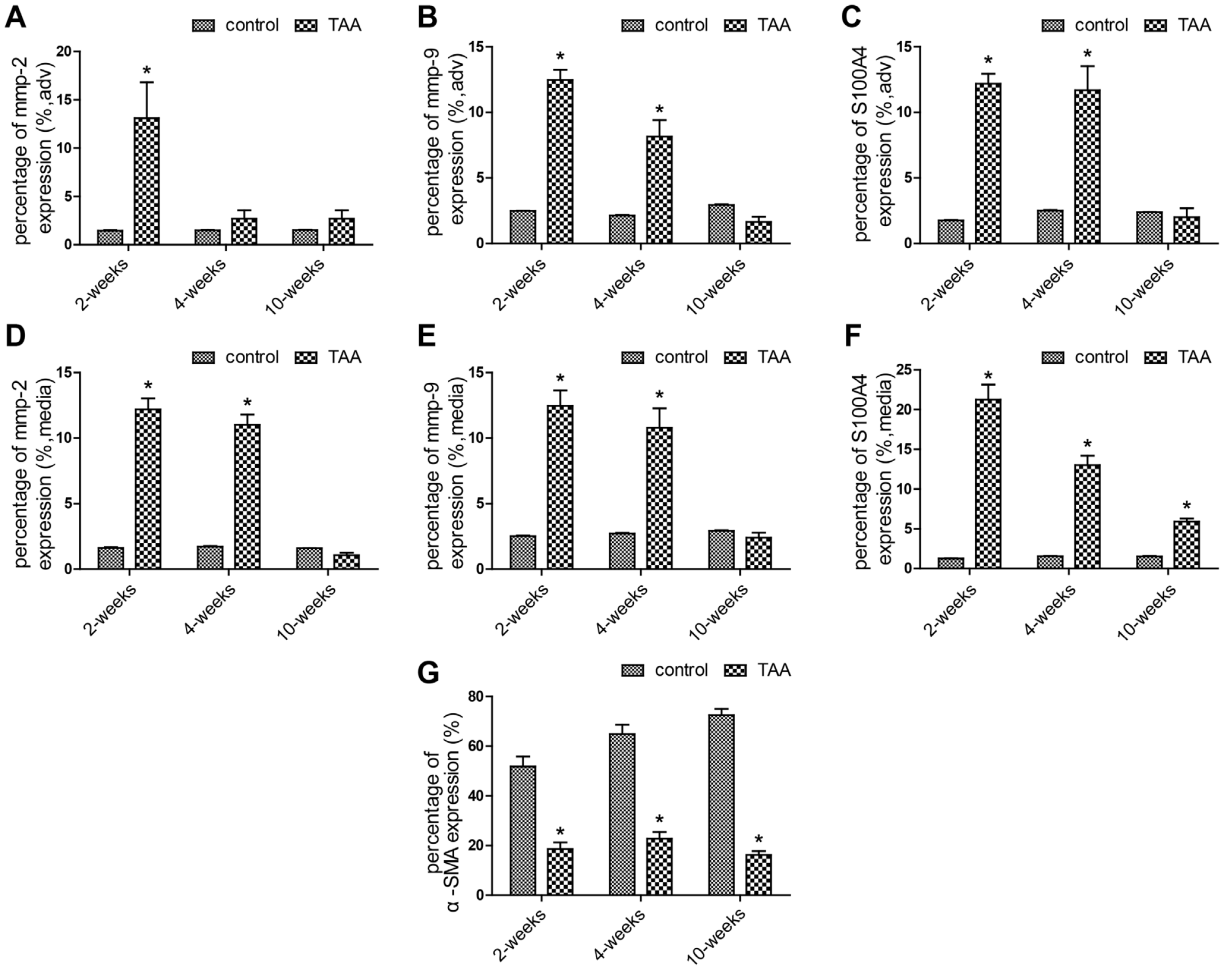
Quantitation of the protein content of MMP-2, MMP-9, S100A4 and αSMA in aortic slides over time post-TAA induction. The immunohistochemically stained slides were assessed by computerized planimetry in the aortic adventitia (A, B and C) and aortic media (D, E, F and G). Bars represent the SEM, *P<0.05 compared with control group (n = 6 for each group).

A similar change in protein expression level was observed at all time-points by immunohistochemistry. In the CaCl_2_-treated segments, MMP-2 was significantly increased in all layers of the aorta at 2 weeks and in the medial layer at 4 weeks, while NaCl-treated segments only showed mild staining **(** **Fig. 4** **)**. MMP-9 protein was found at peak level in the adventitia and elastic lamellae at 2 weeks, but decreased at 4 weeks and later. The temporal change in the S100A4 protein level was consistent with the expression of MMP-2 and MMP-9. A significant increase in S100A4 protein was observed at 2 and 4 weeks, compared to the low staining observed in controls **(** **Fig. 4** **)**. In the vessel wall, αSMA was significantly decreased across all time points in comparison to the NaCl-treated group (**Fig. 4** **)**. Quantitation of the protein content of MMP-2, MMP-9, S100A4 and αSMA were assessed by computerized planimetry in the aortic adventitia and aortic media in immunohistochemically stained slides **(** **Fig. 5** **)**.

**Figure 6:**
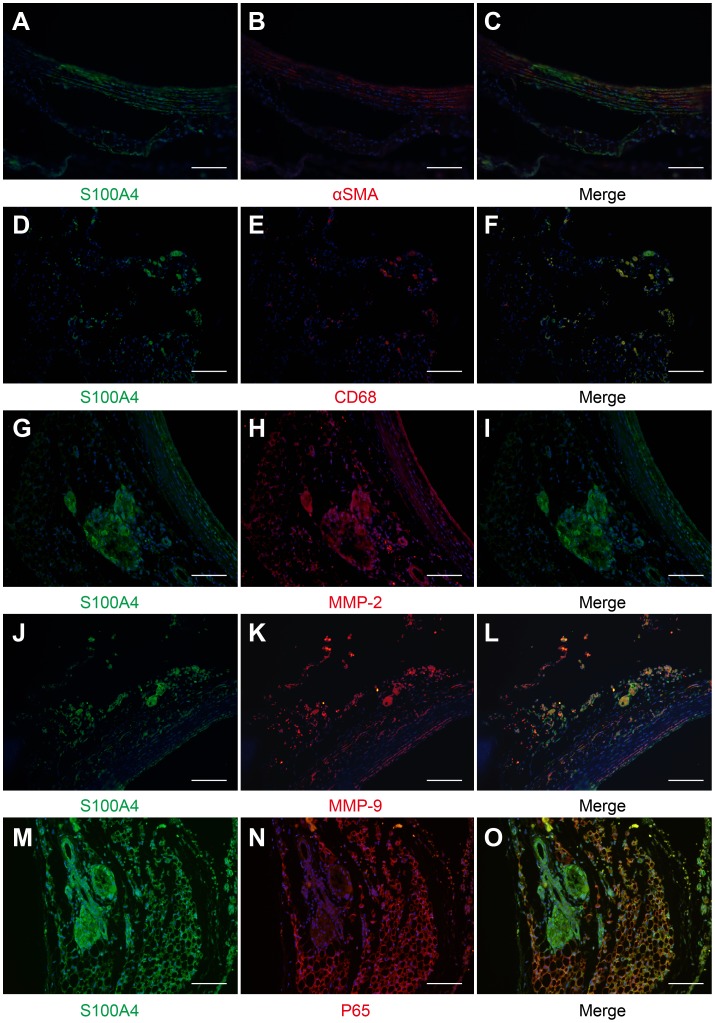
Immunofluorescent staining of S100A4 and the other specific cell markers in aortic slides 4 weeks post-TAA induction. Representative images of co-staining of S100A4 (FITC-conjugated green, A, D, G, J and M) with αSMA (Alexa Fluor 594-conjugated red, B), CD68 (red, E), MMP-2 (red, H), MMP-9 (red, K) and P65 (red, N) in the CaCl_2_-treated segment (4 weeks post-TAA induction). The yellow staining denotes colocalization (C, F, I, L and O). Scale bar = 50 µm.

Mesenchymal cells and macrophages have been shown to be the two main sources of MMPs in the process of aneurysmal remodeling (Longo et al., 2002). Since chronic inflammation mediated by macrophages in vascular walls may contribute to the aneurysmal remodeling, to clarify the potential involvement of S100A4 in the remodeling process, immunofluorescent staining was performed on S100A4 along with the other specific cell markers of macrophage-mediated inflammatory pathways. S100A4 was strongly expressed in CD68+cells, whereas αSMA-positive labeled cells were only partially stained with S100A4 (**Fig. 6**). In addition, co-staining with MMP2, MMP9 and NF-κB P65 subunit confirmed the maximal expression of S100A4 at sites of inflammation and destruction **(** **Fig. 6** **)**.

### Down-regulation of S100A4 Inhibits Cell Proliferation of A7r5 and MMP2/MMP9 Expression *in vitro*

**Figure 7:**
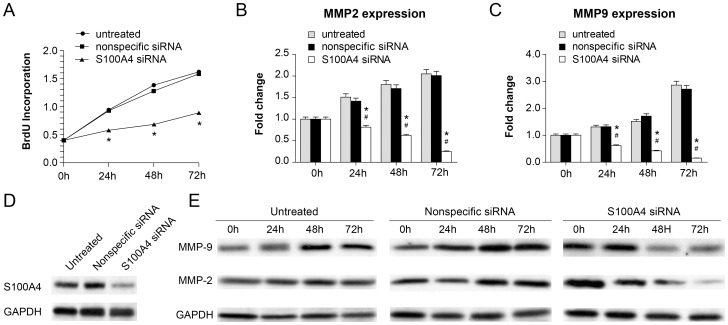
Down-regulation of S100A4 inhibited cell proliferation of A7r5 and MMP2/MMP9 expression *in vitro*. A, The results of cell proliferation ability of A7r5 cells after transfection with S100A4 siRNA at different time intervals (0, 24, 48, 72 h). *Represents *P*<0.05, compared to the control nonspecific siRNA-transfected group and the untreated A7r5 group at 24, 48, and 72 h. B, RT-PCR analysis of MMP2 mRNA isolated from A7r5 cells from 3 groups after transfection at different time intervals (0, 24, 48, 72 h). * and # represents *P*<0.05, compared to the control nonspecific siRNA-transfected group and the untreated A7r5 group at 24, 48, and 72 h. C, RT-PCR analysis of MMP9 mRNA isolated from A7r5 cells from 3 groups after transfection at different time intervals (0, 24, 48, 72 h). * and # represents *P*<0.05, compared to the control nonspecific siRNA-transfected group and the untreated A7r5 group at 24, 48, and 72 h. D, Western blot analysis of the knockdown efficiency of S100A4 by siRNA. E, Western blot analysis of MMP2/MMP9 protein isolated from A7r5 cells from 3 groups after transfection at different time intervals (0, 24, 48, 72 h).

The A7r5 cell line is an adherent smooth muscle cell line derived from rat aortic fibroblasts. Because of the similarity in the spatiotemporal expression pattern between MMPs and S100A4, we used siRNA technology to knock down S100A4 expression in A7r5 cells to test the hypothesis that S100A4 functions as a regulator of MMP expression. As shown in **Fig. 7**, S100A4-specific siRNA transfection led to impaired proliferation of the A7r5 cells compared to untreated cells or cells transfected with non-specific siRNA. Remarkably, suppression of S100A4 expression appeared to inhibit MMP2 and MMP9 expression, indicating S100A4 does function to regulate MMP2 and MMP9 expression. Since previous studies revealed that MMP2 gene expression is trans-activated by P53 through the consensus P53 binding site in the MMP2 promoter region [12], [13], we speculated that the P53 gene might be involved in the S100A4-MMP2/MMP9 regulatory pathway.

### S100A4 Expression in Human TAA Tissue and Blood

**Figure 8:**
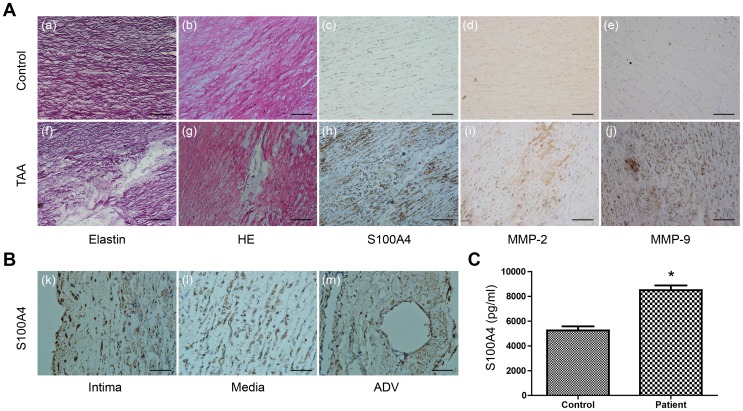
S100A4 expression in human TAA tissue and blood. A, Representative images of morphological and immunohistochemical analysis of a human TAA sample. The slides were stained with Weigert solution (a) (f), hematoxylin/eosin (b) (g), anti-S100A4 antibody (c) (h), anti-MMP-2 antibody (d) (i) and anti-MMP-9 antibody (e) (j). Scale bar = 100 µm. B, Higher power micrographs of the localization of S100A4 in the aortic intima (k), media (l) and adventitia (m). Scale bar = 50 µm. C, Concentrations of S100A4 determined by ELISA in serum obtained from TAA patients (n = 6) and control patients (n = 6). Values are the means ± SEM. *P<0.05, indicates a significant difference from control patients.

We further investigated S100A4 expression in aortic tissues collected from human TAA patients. As shown in **Fig. 8A**, increased expression of S100A4 was observed in the αSMA-positive cells across the entire aortic wall of aneurysm samples, with the medial layer being more prominent than the intima and adventitia, results which resemble the findings in the animal model. HE staining displayed zonal necrosis of the medial layer, accumulation of mucopolysaccharides, and focal loss of vascular smooth muscle cells. In addition, focal elastic fiber loss and fragmentation were also typical features of aortic aneurysm. Notably, we also found that compared to the control samples, TAA patients (n = 6) also carried significantly higher S100A4 concentrations in the serum **(** **Fig. 8C** **)**. Clinical characteristics of 6 TAA patients and 6 control patients are described in **table 1**.

**Table 1:** Clinical Characteristics of TAA Patients and Control Patients.

# of cases	Age/Gender	Clinical phenotype
TAA patients		
1	30 yr/F	Thoracic aortic aneurysm distal to the left subclavin extending to the diaphragmatic crura,the widest transverse diameter measuring 6.8cm
2	34 yr/M	Thoracic aortic aneurysm extending to the abdominal aorta with a length of 7.0 cm and a diameter of 4.1 cm
3	55 yr/M	Ascending thoracic aortic aneurysm, the patient died of rupture
4	35 yr/M	Descending thoracic aortic aneurysm with a diameter of 5.4 cm
5	39 yr/F	Thoracic aortic aneurysm with a diameter of 4.9 cm, with Marfan Syndrom
6	68 yr/M	Ascending thoracic aortic aneurysm with a diameter of 5.7 cm
Control patients		
1	35 yr/F	rupture of oophoritic cyst
2	30 yr/M	traffic accident
3	58 yr/M	Mallory-Weiss Syndrome
4	39 yr/M	acute necrotizing pancreatitis
5	36 yr/F	rupture of ectopic pregnancy
6	70 yr/M	fracture of the hip joint

## Discussion

S100A4 belongs to the S100 calcium-binding protein family and functions as a small, acidic calcium-binding protein transducing Ca^2+^-signals via interaction with intracellular target proteins [14]. It is well recognized that Ca^2+^signaling pathways play critical roles in a wide range of physiological functions including control and regulation of smooth muscle cell performance and vascular tone, and that deregulation of S100 proteins would lead to profound effects on the Ca^2+^signaling systems with detrimental consequences for cardiac and skeletal muscle, and vascular function. To date, S100A4 has been mainly known as a cancer metastasis gene and its expression has been shown to promote cancer metastasis by inducing angiogenesis and disruption of the extracellular matrix [15]. However, previous studies have indicated that S100A4 can increase secretion of MMPs from endothelial cells and fibroblasts [16], [17], and that it is highly expressed in smooth muscle cells in atherosclerotic lesions and pulmonary vascular diseases [18], [19]. Taken together, these data suggest a critical impact of increased S100A4 levels on cellular function, inflammation and extracellular matrix turnover in malignant or non-malignant pathology. However, whether S100A4 is related to TAA formation remains unknown.

MMPs are a family of zinc-dependent endopeptidases capable of degrading different extracellular matrix components including collagens and elastin. Under normal physiological conditions, the enzymatic activity of MMPs is tightly controlled by their inhibitors and seldom detectable, but in vascular pathologies (such as aneurysm, stenosis, or atherosclerosis), increased expression of MMPs has often been observed. During formation of an aneurysm, infiltrating inflammatory cells (mainly macrophages), and smooth muscle cells, fibroblasts and endothelial cells from blood vessels can all produce MMPs, leading to degradation of the extracellular matrix and remodeling of the artery walls [20]. In the current study, by investigating the spatiotemporal expression of S100A4 and two MMP genes (MMP2 and MMP9) in CaCl_2_-induced rat TAA aortic tissues, we identified a significant correlation between MMP2, MMP9 and S100A4 expression in the process of TAA formation. Such remarkable similarity in the expression patterns prompted us to speculate that S100A4 may be functionally involved in the regulation of MMP expression. Indeed, effects of S100A4 on regulation of MMPs have been noted in several previous experiments studying its pro-metastatic properties [15]. In this study, the *in vitro* siRNA transfection assay demonstrated that knock-down of S100A4 expression could lead to inhibition of MMP2 expression, thus providing direct evidence that S100A4 can contribute to TAA pathogenesis, at least partially through MMP proteins.

S100A4 is widely expressed in tissue resident cells and infiltrated inflammatory cells in any chronically inflamed tissue with increased turnover of extracellular matrix [21]. In the current study, we observed that S100A4/MMP-expressing cells are also CD68-and NF-κB P65-positive, suggesting that these cells also possess inflammatory properties. The S100 family comprises more than 20 calcium-binding proteins. Physiologically, they exert numerous autocrine and paracrine functions on cellular proliferation, differentiation and cell motility [22]. It has been shown that S100A1 overexpression enhances cardiac contractile performance, establishing the concept of S100A1 as a regulator of myocardial contractility [23]. S100/calgranulins, including S100A8 (Calgranulin A), S100A9 (Calgranulin B) and S100A12 (Calgranulin C), are expressed predominantly in neutrophils and macrophages, and play a regulatory role in the inflammatory cascade [24]. Like calgranulins, S100A4 has been suggested as a pro-inflammatory factor, due to its contribution to proliferation, inflammatory angiogenesis, and extracellular matrix remodeling [11], [16]. Previous studies have demonstrated the roles of calgranulins and S100A4 in the pathogenic process of several autoimmune disorders including rheumatoid arthritis, inflammatory bowel diseases, and others [25]. Our current result provides novel information about extracellular matrix remodeling by linking S100A4 to TAA, and supports the hypothesis that aortic aneurysm and autoimmune disease share common degenerative features [26].

Our studies on human TAA patients revealed that S100A4 expression is also up-regulated in diseased aortic tissues. This significant increase in the serum concentration of S100A4 in TAA patients compared to healthy individuals means that with more thorough investigation, S100A4 expression could become a potential biomarker for diagnosis and treatment of TAA in future. In summary, the present study provided unique insights into the mechanisms of TAA formation and suggests that therapeutic treatments targeting S100A4 can potentially be beneficial to the management of patients with TAA.

## Supporting Information

Table S1 Antibodies and dilutions.
(DOCX)

## References

[1] Lindsay ME, Dietz HC (2011) Lessons on the pathogenesis of aneurysm from heritable conditions. Nature 473: 308–316. 21593863 10.1038/nature10145 PMC3622871

[2] Milewicz DM, Guo DC, Tran-Fadulu V, Lafont AL, Papke CL, et al (2008) Genetic basis of thoracic aortic aneurysms and dissections: focus on smooth muscle cell contractile dysfunction. Annu Rev Genomics Hum Genet 9: 283–302. 18544034 10.1146/annurev.genom.8.080706.092303

[3] Cao J, Gong L, Guo DC, Mietzsch U, Kuang SQ, et al (2010) Thoracic aortic disease in tuberous sclerosis complex: molecular pathogenesis and potential therapies in Tsc2+/− mice. Hum Mol Genet 19: 1908–1920. 20159776 10.1093/hmg/ddq066 PMC2860890

[4] Guo DC, Pannu H, Tran-Fadulu V, Papke CL, Yu RK, et al (2007) Mutations in smooth muscle alpha-actin (ACTA2) lead to thoracic aortic aneurysms and dissections. Nat Genet 39: 1488–1493. 17994018 10.1038/ng.2007.6

[5] Segura AM, Luna RE, Horiba K, Stetler-Stevenson WG, McAllister HA, Jr., et al.. (1998) Immunohistochemistry of matrix metalloproteinases and their inhibitors in thoracic aortic aneurysms and aortic valves of patients with Marfan’s syndrome. Circulation 98: II331–337; discussion II337–338. 9852923

[6] Jones JA, Barbour JR, Lowry AS, Bouges S, Beck C, et al (2006) Spatiotemporal expression and localization of matrix metalloproteinas-9 in a murine model of thoracic aortic aneurysm. J Vasc Surg 44: 1314–1321. 17145436 10.1016/j.jvs.2006.07.042 PMC1761919

[7] Geng L, Wang W, Chen Y, Cao J, Lu L, et al (2010) Elevation of ADAM10, ADAM17, MMP-2 and MMP-9 expression with media degeneration features CaCl2-induced thoracic aortic aneurysm in a rat model. Exp Mol Pathol 89: 72–81. 20621845 10.1016/j.yexmp.2010.05.006

[8] Longo GM, Xiong W, Greiner TC, Zhao Y, Fiotti N, et al (2002) Matrix metalloproteinases 2 and 9 work in concert to produce aortic aneurysms. J Clin Invest 110: 625–632. 12208863 10.1172/JCI15334 PMC151106

[9] Xiong W, Knispel RA, Dietz HC, Ramirez F, Baxter BT (2008) Doxycycline delays aneurysm rupture in a mouse model of Marfan syndrome. J Vasc Surg 47: 166–172; discussion 172. 10.1016/j.jvs.2007.09.016 PMC4148046 18178469

[10] Chung AW, Yang HH, Radomski MW, van Breemen C (2008) Long-term doxycycline is more effective than atenolol to prevent thoracic aortic aneurysm in marfan syndrome through the inhibition of matrix metalloproteinase-2 and -9. Circ Res 102: e73–85. 18388324 10.1161/CIRCRESAHA.108.174367

[11] Boye K, Maelandsmo GM (2010) S100A4 and metastasis: a small actor playing many roles. Am J Pathol 176: 528–535. 20019188 10.2353/ajpath.2010.090526 PMC2808059

[12] Barak Y, Juven T, Haffner R, Oren M (1993) mdm2 expression is induced by wild type p53 activity. EMBO J 12: 461–468. 8440237 10.1002/j.1460-2075.1993.tb05678.x PMC413229

[13] Bian J, Sun Y (1997) Transcriptional activation by p53 of the human type IV collagenase (gelatinase A or matrix metalloproteinase 2) promoter. Mol Cell Biol 17: 6330–6338. 9343394 10.1128/mcb.17.11.6330 PMC232484

[14] Engelkamp D, Schafer BW, Erne P, Heizmann CW (1992) S100 alpha, CAPL, and CACY: molecular cloning and expression analysis of three calcium-binding proteins from human heart. Biochemistry 31: 10258–10264. 1384693 10.1021/bi00157a012

[15] Bjornland K, Winberg JO, Odegaard OT, Hovig E, Loennechen T, et al (1999) S100A4 involvement in metastasis: deregulation of matrix metalloproteinases and tissue inhibitors of matrix metalloproteinases in osteosarcoma cells transfected with an anti-S100A4 ribozyme. Cancer Res 59: 4702–4708. 10493528

[16] Senolt L, Grigorian M, Lukanidin E, Simmen B, Michel BA, et al (2006) S100A4 is expressed at site of invasion in rheumatoid arthritis synovium and modulates production of matrix metalloproteinases. Ann Rheum Dis 65: 1645–1648. 17105852 10.1136/ard.2005.047704 PMC1798462

[17] Schmidt-Hansen B, Ornas D, Grigorian M, Klingelhofer J, Tulchinsky E, et al (2004) Extracellular S100A4(mts1) stimulates invasive growth of mouse endothelial cells and modulates MMP-13 matrix metalloproteinase activity. Oncogene 23: 5487–5495. 15122322 10.1038/sj.onc.1207720

[18] Brisset AC, Hao H, Camenzind E, Bacchetta M, Geinoz A, et al (2007) Intimal smooth muscle cells of porcine and human coronary artery express S100A4, a marker of the rhomboid phenotype in vitro. Circ Res 100: 1055–1062. 17347479 10.1161/01.RES.0000262654.84810.6c

[19] Greenway S, van Suylen RJ, Du Marchie Sarvaas G, Kwan E, Ambartsumian N, et al (2004) S100A4/Mts1 produces murine pulmonary artery changes resembling plexogenic arteriopathy and is increased in human plexogenic arteriopathy. Am J Pathol 164: 253–262. 14695338 10.1016/S0002-9440(10)63115-X PMC1602221

[20] Zhang X, Shen YH, LeMaire SA (2009) Thoracic aortic dissection: are matrix metalloproteinases involved? Vascular 17: 147–157. 19476747 10.2310/6670.2008.00087 PMC2843550

[21] Klingelhofer J, Senolt L, Baslund B, Nielsen GH, Skibshoj I, et al (2007) Up-regulation of metastasis-promoting S100A4 (Mts-1) in rheumatoid arthritis: putative involvement in the pathogenesis of rheumatoid arthritis. Arthritis and rheumatism 56: 779–789. 17328050 10.1002/art.22398

[22] Heizmann CW, Fritz G, Schafer BW (2002) S100 proteins: structure, functions and pathology. Front Biosci 7: d1356–1368. 11991838 10.2741/A846

[23] Most P, Bernotat J, Ehlermann P, Pleger ST, Reppel M, et al (2001) S100A1: a regulator of myocardial contractility. Proc Natl Acad Sci U S A 98: 13889–13894. 11717446 10.1073/pnas.241393598 PMC61137

[24] Pietzsch J, Hoppmann S (2009) Human S100A12: a novel key player in inflammation? Amino Acids 36: 381–389. 18443896 10.1007/s00726-008-0097-7

[25] Oslejskova L, Grigorian M, Gay S, Neidhart M, Senolt L (2008) The metastasis associated protein S100A4: a potential novel link to inflammation and consequent aggressive behaviour of rheumatoid arthritis synovial fibroblasts. Ann Rheum Dis 67: 1499–1504. 18056757 10.1136/ard.2007.079905

[26] Kuivaniemi H, Platsoucas CD, Tilson MD, 3rd (2008) Aortic aneurysms: an immune disease with a strong genetic component. Circulation 117: 242–252. 18195185 10.1161/CIRCULATIONAHA.107.690982 PMC3001294

