# Supplementary material for: Spatiotemporal Expression of Matrix Metalloproteinases (MMPs) is Regulated by the Ca2+-Signal Transducer S100A4 in the Pathogenesis of Thoracic Aortic Aneurysm
Source: PLoS One. 2013 Jul 29;8(7):e70057. doi: 10.1371/journal.pone.0070057 (PMC3726393; doi:10.1371/journal.pone.0070057)
Supplement: Table S1 — Antibodies and dilutions. (DOCX) [file pone.0070057.s001.docx]

| **Table S1: Antibodies and dilutions.** | | | |
| --- | --- | --- | --- |
| **Primary antibodies** | | | |
| **antibody** | **company** | **species** | **IHC** |
| MMP-2 | Cell Signal | rabbit | 1:100 |
| MMP-9 | Cell Signal | rabbit | 1:100 |
| SM α-actin | Sigma | mouse | 1:200 |
| P-65 | Santa Cruz | mouse | 1:100 |
| CD3 | Abcam | rabbit | 1:200 |
| CD68 | Abcam | rabbit | 1:200 |
| S100A4 | Abcam | rabbit | 1:400 |
